# An international phantom study of inter-site variability in Technetium-99m image quantification: analyses from the TARGET radioembolization study

**DOI:** 10.1186/s40658-024-00647-x

**Published:** 2024-05-29

**Authors:** Grace Keane, Rob van Rooij, Marnix Lam, S. Cheenu Kappadath, Bilal Kovan, Stephanie Leon, Matthew Dreher, Kirk Fowers, Hugo de Jong

**Affiliations:** 1 Department of Radiology and Nuclear Medicine, University Medical Center Utrecht, 3508 GA Utrecht, The Netherlands; 2 Department of Interventional Radiology, University of Texas MD Anderson Cancer Center, Houston, TX USA; 3 Department of Nuclear Medicine, Istanbul Faculty of Medicine, Istanbul University, Istanbul, Turkey; 4 Department of Radiology, University of Florida, Gainesville, FL USA; 5 Boston Scientific Corporation, Marlborough, MA USA

**Keywords:** Technetium-99m, Macroaggregated-albumin (MAA), Imaging, Performance, SPECT/CT, Harmonization, Yttrium-90

## Abstract

**Background:**

Personalised multi-compartment dosimetry based on [^99m^Tc]Tc-MAA is a valuable tool for planning ^90^Y radioembolization treatments. The establishment and effective application of dose–effect relationships in yttrium-90 (^90^Y) radioembolization requires [^99m^Tc]Tc-MAA SPECT quantification ideally independent of clinical site. The purpose of this multi-centre phantom study was to evaluate inter-site variability of [^99m^Tc]Tc-MAA imaging and evaluate a standardised imaging protocol. Data was obtained from the TARGET study, an international, retrospective multi-centre study including 14 sites across 8 countries. The impact of imaging related factors was estimated using a NEMA IQ phantom (representing the liver), and a uniformly filled cylindrical phantom (representing the lungs). Imaging was performed using site-specific protocols and a standardized protocol. In addition, the impact of implementing key image corrections (scatter and attenuation correction) in the site-specific protocols was investigated. Inter-site dosimetry accuracy was evaluated by comparing computed Lung Shunt Fraction (LSF) measured using planar imaging of the cylindrical and NEMA phantom, and contrast recovery coefficient (CRC) measured using SPECT imaging of the NEMA IQ phantom.

**Results:**

Regarding the LSF, inter-site variation with planar site-specific protocols was minimal, as determined by comparing computed LSF between sites (interquartile range 9.6–10.1%). A standardised protocol did not improve variation (interquartile range 8.4–9.0%) but did improve mean accuracy compared to the site-specific protocols (5.0% error for standardised protocol vs 8.8% error for site-specific protocols). Regarding the CRC, inter-system variation was notable for site-specific SPECT protocols and could not be improved by the standardised protocol (CRC interquartile range for 37 mm sphere 0.5–0.7 and 0.6–0.8 respectively), however the standardised protocol did improve accuracy of sphere:background determination. Implementation of key image corrections did improve inter-site variation (CRC interquartile range for 37 mm sphere 0.6–0.7).

**Conclusion:**

Eliminating sources of variability in image corrections between imaging protocols reduces inter-site variation in quantification. A standardised protocol was not able to improve consistency of LSF or CRC but was able to improve accuracy.

**Supplementary Information:**

The online version contains supplementary material available at 10.1186/s40658-024-00647-x.

## Introduction

Radioembolization with yttrium-90 (^90^Y) (TheraSphere™, Boston Scientific Corporation, Marlborough, MA, USA) glass microspheres is a well-established locoregional treatment option for hepatocellular carcinoma (HCC) [1]. In this procedure, glass microspheres of diameter 15–35 μm containing radioactive ^90^Y, are administered to liver tumours via catheterization of the hepatic artery. Since the hepatic artery almost exclusively supplies tumour vasculature, the microspheres are flushed into the target tissue where they lodge within tumour arterioles and deliver a localised radiotherapeutic dose via emitted beta radiation. The targeted administration of the microspheres facilitates an optimised dose distribution, where tumours are maximally dosed, and normal tissue largely spared.

Studies evaluating the relationship of dose and clinical outcomes demonstrate that, where personalised dosing is used to achieve a maximised tumour absorbed dose, outcomes (including response and survival) are optimised [2–4]. Personalised dosimetry may be implemented by using a pre-treatment scintigraphy (planar, SPECT or SPECT/CT) study, from which anticipated tumour absorbed dose, normal tissue dose and lung dose can be calculated for each individual. Single-photon emission computed tomography (SPECT) and computed tomography (CT) hybrid imaging (SPECT/CT) using ^99m^Tc-macroaggregated-albumin (MAA) is the accepted standard for the pre-treatment or ‘scout’ procedure, which forms the basis of predictive dosimetry calculations. It has been widely evidenced that [^99m^Tc]Tc-MAA is a viable surrogate agent [5, 6] and is consequently recommended in best practice guidelines as an essential step in the ^90^Y treatment workup [1].

Tumour absorbed dose and lung shunt fraction are two of the most frequently cited quantitative metrics derived from multi-compartment analysis of [^99m^Tc]Tc-MAA SPECT/CT [7] and represent important indicators of the expected efficacy and safety of the therapy. However, quantitative values in [^99m^Tc]Tc-MAA SPECT/CT can exhibit variability due to technical factors including; gamma ray attenuation in the patient, scatter in the patient and detector, imaging system resolution (partial volume effects), and image noise [8–11]. This can substantially impact quantitative accuracy. Comparable performance that allows for reliable comparison of quantitative values between systems is essential in multi-centre studies. To leverage [^99m^Tc]Tc-MAA SPECT/CT as a dosimetry tool with minimal bias and variability, harmonization strategies must be implemented.

The TheraSphere Advanced Dosimetry Retrospective Global Study Evaluation in Hepatocellular Carcinoma Treatment (TARGET) study focused on the exploration of multi-compartment dosimetry based on [^99m^Tc]Tc-MAA SPECT/CT in an international, retrospective multi-centre study [12]. Images were generated independently by each participating centre, and therefore a range of different imaging devices and protocols were used in the generation of the dataset. A broad spectrum of SPECT/CT systems were included, ranging from first generation scanners to those incorporating contemporary technologies with advanced acquisition and reconstruction capabilities. Similarly, a range of imaging protocols were included with varying acquisition and reconstruction parameters. These protocols made reference to international guidelines [13] and so shared some commonalities, but were largely distinct.

Conducting inter-site comparisons between centres using different devices, and where there was no uniform approach to imaging procedures, results in variability which must be characterised. In addition, widespread clinical application of the resulting dose thresholds beyond the study requires a harmonized method to quantify the SPECT images.

The aim of this phantom study was twofold; firstly to use multicentre phantom data to evaluate inter-site variability of dosimetry based on [^99m^Tc]Tc-MAA imaging, and secondly, to implement and evaluate a standardised imaging protocol.

## Materials and methods

Phantom images were used to identify the impact of acquisition and reconstruction related factors on dosimetry via measurement of simulated Lung Shunt Fraction (LSF) and tumour to normal tissue (T:N) ratio (evaluated via Contrast Recovery Coefficient (CRC)).

### Phantom preparation

Each participating centre was provided with two phantoms; a National Electrical Manufacturers Association (NEMA) NU-2 image quality (IQ) phantom (Data Spectrum Corporations—NC) and a hollow cylinder (diameter 20 ± 2 cm).

#### NEMA IQ phantom

For the liver model a NEMA IQ phantom compliant with NU2 2007 standards [14] was utilised, which contained 6 spheres (representing tumoral liver) of diameter 10, 13, 17, 22, 28 and 37 mm and a cylindrical insert of diameter 50 mm inside a 9.7 L background volume (representing non-tumoral liver). Spherical inserts were filled with [^99m^Tc]Tc-pertechnetate of activity concentration 0.24 MBq/ml, and the background volume with a concentration of 0.03 MBq/ml, such that there was an approximate 8:1 sphere to background ratio and a total activity of 300 MBq, per the TARGET protocol. The selected activities exceed those typically used clinically, due to the substantially larger volume of the NEMA phantom compared to a standard liver.

#### Cylindrical phantom

For the lung model a cylindrical phantom of diameter 20 ± 2 cm was utilised, with length sufficient to cover the axial field of view (FOV). The exact dimensions and volume of the phantom was recorded by each site. The phantom was filled with water and 30 MBq added, such that there was an approximate 10:1 ratio between the NEMA phantom activity and cylindrical phantom activity.

Each site provided details of injected activity, residual activity, volume measurements, date/time of measurements and dose calibrator information.

### Phantom positioning

**Fig. 1:**
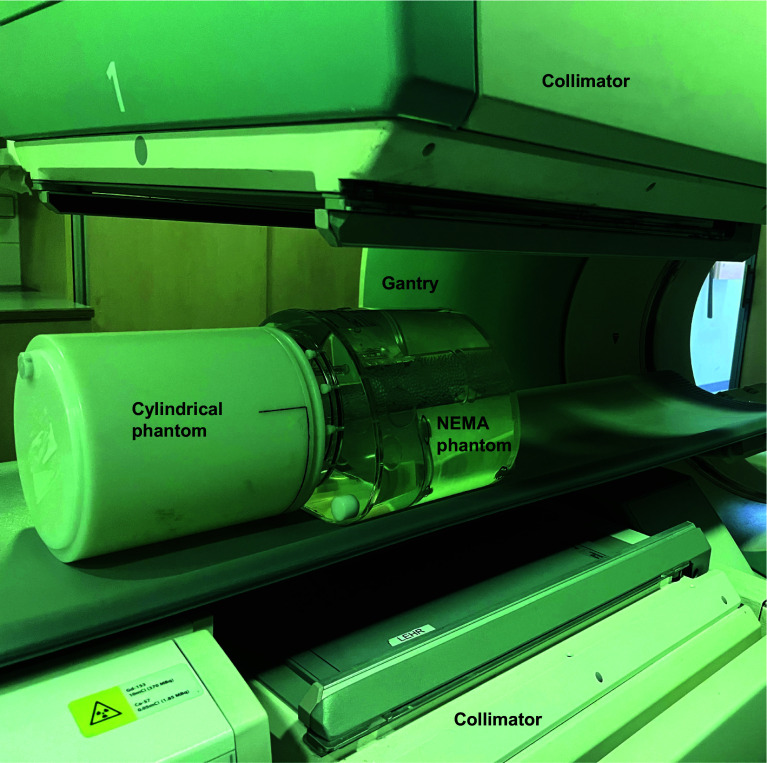
NEMA and cylindrical phantom positioned for a feet-first protocol

The phantoms were positioned adjacent to each other on the scanner couch (Fig. 1). The relative positioning of the phantoms was dependent on the given sites acquisition protocol and could be either feet-first or head-first.

### Phantom imaging

#### Site-specific protocols

Each participating centre was asked to perform planar and SPECT acquisitions based on their site-specific clinical protocol used for pre-treatment [^99m^Tc]Tc-MAA imaging. Planar images of the cylindrical phantom (representing the lungs) and NEMA IQ phantom (representing the liver) were used in the LSF evaluation. SPECT images of the NEMA IQ phantom were used for CRC evaluation. If patient data included in the TARGET clinical study was generated with more than one imaging protocol (i.e. an old and updated protocol), imaging was to be repeated for every protocol that had been utilised to scan patients.

#### Standardised protocol

Centres were then asked to perform a second acquisition and reconstruction using a standardized protocol (parameters shown in Table 1), which was identical for every centre. Reconstructions were centrally reviewed to confirm whether they matched the standard protocol. If a participating centre was unable to fully apply the standardized protocol due to technical reasons, a protocol deviation was noted within the final dataset, and they were excluded. The TARGET phantom study standardised acquisition and reconstruction protocol was designed to ensure widespread utility on a variety of scanner types and was not optimised for lesion detection or image quality.

**Table 1:** Acquisition and reconstruction parameters for the TARGET standardised protocol

Parameter	Standardised protocol Criteria
Hybrid SPECT/CT (yes/no)	Yes
Collimator type and name	Low Energy High Resolution (LEHR)
Energy window; centre (keV) and range (%)	140.5 keV/15%
Scatter window	Dual Energy Window (DEW) 15%
*Planar*	
Number of acquisitions	2
Acquisition time per frame (min.)	5
Zoom	1
Matrix size	256 × 256
Additional information	First acquisition centred on lung, second on liver
Scatter correction	Yes (centrally applied)
Geometric mean	Yes
*SPECT*	
Matrix size	128 × 128
Zoom	1.0
Number of heads	2
Number of projections	120
Time per projections(sec.)	15
Orbit	Non-circular close orbit
*SPECT reconstruction*	
Type	Ordered subset expectation maximization (OS-EM)
Matrix	128 × 128x128
Iterations (OSEM)	20
Subsets (OSEM)	8
Attenuation correction	CT based attenuation map
Scatter correction	DEW
Other (partial volume etc.)	None
Filtering	5 mm Gaussian

### Data collection

Each participating centre was required to submit full activity measurements and dose calibrator information, as well as a complete description of the site-specific planar and SPECT acquisition protocols (field of view positioning, acquisition parameters etc.) and site-specific reconstruction protocols (attenuation correction, scatter correction, point spread function modelling).

Planar data, SPECT projection data and reconstructed SPECT images generated with the site-specific and standardised protocol respectively were provided by each centre, together with the co-registered CT scan.

### Image analysis

#### Lung shunt fraction investigation

The LSF was measured based on planar images, which is standard in clinical practice. Regions of interest (ROIs) were drawn around the liver and lungs using the NEMA IQ phantom and cylindrical phantom, respectively. The LSF was calculated as a percentage based on the counts identified in these ROIs, using Eq. 1.

1 $$LSF = \frac{{Counts_{Lung} }}{{Counts_{Liver} + Counts_{Lung} }}$$

ROIs were defined automatically according to the following steps: a scatter window was subtracted from the photopeak window using a k-factor of 0.5 and corrected to account for the possible difference in window widths. A geometric mean image was constructed by blurring the anterior and posterior views, multiplying them, and taking the square root on a pixel-by-pixel basis. ROIs were automatically defined around the liver phantom and the lung phantom separately [15]. Both ROIs were dilated to ensure all counts were included. Scatter correction was retrospectively applied to data acquired using the standardised protocol centrally.

#### Tumour dosimetry investigation

Contrast recovery coefficients were calculated and regarded as a proxy measure of tumour to normal tissue ratio. SPECT images of the NEMA IQ phantom were analysed by registering a volume of interest (VOI) template over the spherical inserts and in the background compartment. Contrast recovery coefficients were calculated for each sphere size by dividing the measured contrasts by the nominal, true, contrasts determined from the reported injected activities, as shown in Eq. 2.

2 $$CRC = \frac{{C_{s} /C_{B} - 1}}{R - 1}$$

where C_s_ is the mean measured signal in the spherical VOIs, C_B_ the mean measured signal in the background, and R the imposed sphere-to-background activity concentration ratio. Sphere VOIs were defined automatically using a fitting routine in which a template of the spheres was registered to the SPECT and the mean signal in the VOIs was simultaneously optimised. To estimate the background signal, an annular VOI was placed onto a 5 cm stack of slices adjacent to those containing the spheres. This method deviates from the NEMA standard (which stipulates many circular VOIs be placed around the spherical VOIs), however this was deemed necessary as the reconstructed activity distribution in images generated without attenuation correction or scatter correction becomes inhomogeneous with hotter regions near the edge of the phantom, and consequently the background signal would be overestimated.

### Statistical methods

Summary statistics were calculated for the standardised protocol and all site-specific protocols (including from centres with multiple site-specific protocols and excluding data obtained where deviations from the standard protocol were noted), for the endpoints given in Table 2.

**Table 2:** Statistical endpoints

Endpoint	Calculation method
*LSF investigation*	
Lung Shunt Fraction (LSF) % for each scanner and protocol	LSF = counts lung/(counts lung + counts liver)
Difference in measured LSF (%) from the true value relative to the true value, referred to as the LSF error	LSF error = (measured LSF − true value)/true value
*Tumour dosimetry investigation*	
Contrast recovery coefficient (CRC), separately for each insert, for each scanner and protocol	CRC = (measured sphere: background -1)/(true value -1)
Sphere to background (S:B) ratio, separately for each insert, for each scanner and protocol	S:B = measured sphere counts/measured background counts
Difference in measured S:B ratio from the true value, separately for each insert, referred to as the S:B error	S:B error = measured S:B ratio—true value

The analysis of the difference in LSF and CRC between centres constituted a camera-by-camera comparison using a paired t test, where the standardised protocol was compared with the site-specific protocol for the same camera (NB. Only sites which contributed data for the standardised and site-specific subgroups were included in this paired analysis).

Processing of the data was performed centrally, using the Python programming language.

## Results

### General overview

Data was collected over 4 years from 2016 to 2020. A total of 23 SPECT systems (25 site-specific SPECT protocols and 16 site-specific planar protocols) across 14 sites were used for imaging within the TARGET study and included in the current analysis (an overview is given in Table 3). The datasets were 39% (n = 9) from Siemens, 52% (n = 12) from GE and 9% (n = 2) from Philips.

**Table 3:** Summary of SPECT/CT systems and site-specific imaging protocols per system

Site	Model	Planar protocols	SPECT protocols	Attenuation correction (Y/N) / method^1^	Scatter correction (Y/N) / method^2^	Point spread function modelling (Y/N)
250,011	Siemens Symbia T2	✗	✓	Y / (CT)	Y / (DEW)	Y
840,047	GE Infinia	✓	✓*	Y / (CT)	N	N
840,048	GE Discovery 630	✓	✓	Y / (Chang)	N	N
	GE Discovery 670	✓	✓	Y / (CT)	N	N
528,001	Siemens Symbia T	✓*	✓	Y / (CT)	Y / (DEW)	Y
840,098	GE Discovery 670A	✓*	✓*	Y / (CT)	Y / (DEW)	Y
	GE Discovery 670B	✓*	✓*	Y / (CT)	Y / (DEW)	Y
	Philips Brightview XCT	✗	✓*	Y / (CT)	Y / (ESSE)	Y
			✓	Y / (CT)	Y / (ESSE)	N
	Philips Precedence	✓	✓*	Y / (CT)	Y / (ESSE)	Y
			✓	Y / (CT)	Y / (ESSE)	N
756,001	Siemens Intevo	✗	✓*	Y / (CT)	Y / (DEW)	Y
	GE Discovery 670	✗	✓*	Y / (CT)	Y / (DEW)	Y
276,010	Siemens Symbia T2	✓*	✓	Y / (CT)	Y / (DEW)	N
840,017	Encore 2	✗	✓	Y / (Chang)	N	N
840,049	Siemens Symbia T6	✓*	✓*	Y / (CT)	Y / (DEW)	Y
840,051	GE Infinia Hawkeye	✓*	✓✓*	Y / (CT)	Y / (DEW)	Y
	GE Infinia Hawkeye	✓*	✓*	Y / (CT)	Y / (DEW)	Y
792,001	GE Infinia	✓	✓	N	N	Y
	GE Millennium	✓	✓	N	N	N
792,002	GE Discovery 670	✓	✓*	Y / (CT)	Y / (DEW)	N
380,010	GE Infinia		✓	Y / (CT)	Y / (DEW)	Y
	Siemens Intevo		✓	Y / (CT)	Y / (DEW)	Y
840,050	Siemens Intevo	✓*	✓	Y / (CT)	Y / (DEW)	N
	Siemens Symbia	✓*	✓	Y / (CT)	Y / (DEW)	N
Total	23 cameras	16 protocols (9 in paired analysis)	25 protocols (11 in paired analysis)			

✓: Data was submitted for given protocol ✗: Data was submitted for given protocol but could not be analysed (3 × datasets where lung phantom not entirely in field of view, 1 × dataset only anterior images available). *protocols which were considered in the paired analysis

^1^Attenuation correction may be CT based or using Chang’s method

^2^Scatter correction may be dual energy window (DEW) based or using effective scatter source estimation (ESSE)

There were 18 hybrid SPECT/CT scanners, which included a CT scan for attenuation correction (AC), 3 systems were SPECT only and were reliant on a post-registered CT for attenuation correction or Changs [16] correction method, and 2 systems did not supply any attenuation corrected reconstructed data. Scatter correction (SC) was implemented in 19 of the site-specific protocols and 14 implemented point spread function (PSF) modelling (Table 3).

#### Lung shunt fraction investigation

##### Phantom filling

The ratio between activity in the cylindrical ‘lung’ phantom and the NEMA IQ ‘liver’ phantom as required by the protocol was 1:10, corresponding to a ‘true’ lung shunt fraction of 1/(1 + 10): approximately 9.1%. From the reported activities by the participating centres, the injected activities resulted in a median ‘true’ lung shunt fraction of 9.1% (interquartile range (IQR): 8.9%-9.3%). These values are summarised in Table 4, and the raw activity data is given in Supplemental file table S1.

**Table 4:** Summary of true Lung Shunt Fraction (LSF) values based on injected activities for LSF investigation and the measured Lung Shunt Fraction values for the site-specific and standardised protocols

Parameter	True LSF (%)	Site-specific protocol (%)	Standardised protocol (%)
Mean (SD)	9.1 (± 0.52)	9.7 (± 0.53)	8.7 (± 0.57)
Median	9.1	9.9	8.6
Interquartile range	8.9–9.3	9.6–10.1	8.4–9.0

##### Site-specific protocols

After correcting for partial and missing entries and excluding datasets where the phantom was not fully visible in the field of view, data was available for 16 cameras and 16 protocols. Of these, 8 cameras and 8 protocols had both standardised and site-specific data and were used for the paired analysis. Across the 8 considered protocols, the primary emission window was consistent, being centred on 140 keV and with a range of 10–20%. Feet-first positioning was used in 100% of protocols.

The median measured lung shunt fraction for the site-specific protocols was 9.9% (9.6–10.1%) meaning there was a median LSF error of 8.8%

##### Standardised protocol

The median measured LSF for the standard protocol was 8.6% (8.4–9.0%) and LSF error was 5.0%. The LSF values as measured on the site-specific and standardised protocols were significantly different (*p* < 0.001).

**Fig. 2:**
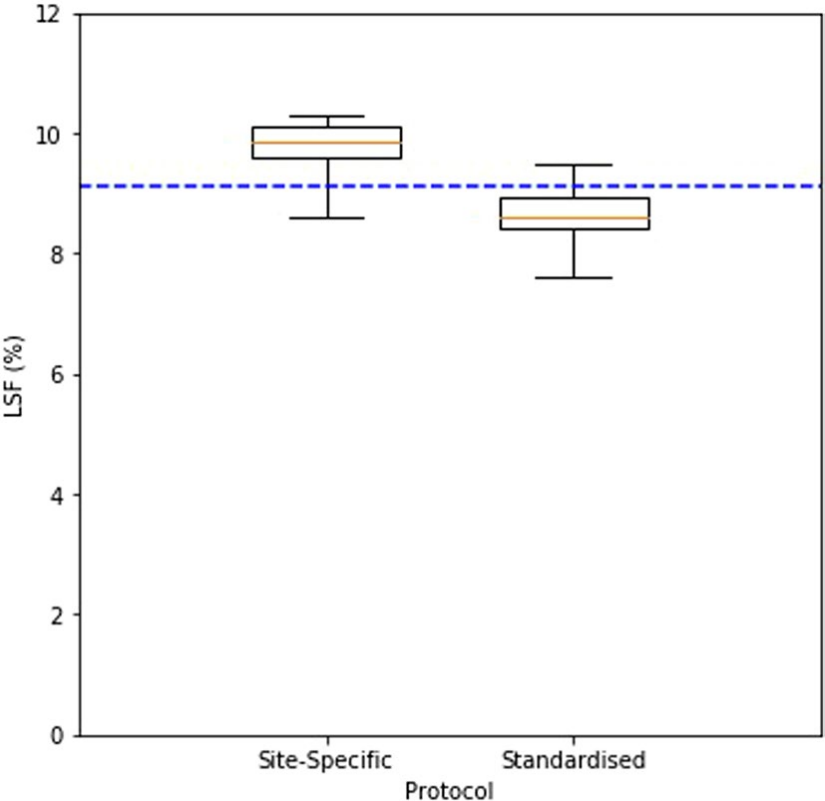
LSF as measured on site-specific protocols and the standardised protocol. Boxplots summarizing the lung shunt fraction values for the site-specific (left) and standardised (right) protocols. The box represents the 25th to 75th percentile range and the central horizontal line represents the median value. The whiskers represent the range. The dashed line represents the true LSF value as determined from injected activities

The median LSF for the site-specific and standardised protocols is shown in Fig. 2 and a summary of measured LSF is given in Table 4.

#### Tumour dosimetry investigation

##### Phantom filling

According to protocol, the intended ratio between activity in the spherical inserts and background compartment was 8:1. From the activities reported by the participating centres, the median ‘true’ sphere to background was 7.9:1 (7.8–8.1). These values are summarised in Table 5, and the raw activity data is given in Supplemental file table S1.

**Table 5:** Summary sphere to background values based on injected activity for tumour dosimetry investigation and the CRC values for the 37 mm sphere for the site-specific and standardised protocols

Parameter	True sphere to background	All site-specific protocols (%)	Site-specific protocols (paired subgroup) (%)		Standardised protocol (%)
Mean (SD)	7.9 (± 0.35)	0.6 (± 0.13)	0.6 (± 0.09)		0.7 (± 0.14),
Median	7.9	0.6	0.6		0.8
Interquartile range	7.8–8.1	0.5–0.7	0.6–0.7		0.6–0.8

##### Site-specific protocols

Data was available for 22 cameras and 25 site-specific protocols, and there were 11 cameras and 11 protocols that supplied both standardised and site-specific data and were used for the paired analysis. Attenuation correction using a co-registered CT was performed in the majority of protocols 20/25 (80%). Scatter correction was less uniformly applied, being performed in 17/25 (68%) of protocols. A full overview of the site-specific protocols is given in Supplemental file table S2. For the paired subgroup, 100% of the protocols used CT based attenuation correction and 90% used scatter correction.

**Fig. 3:**
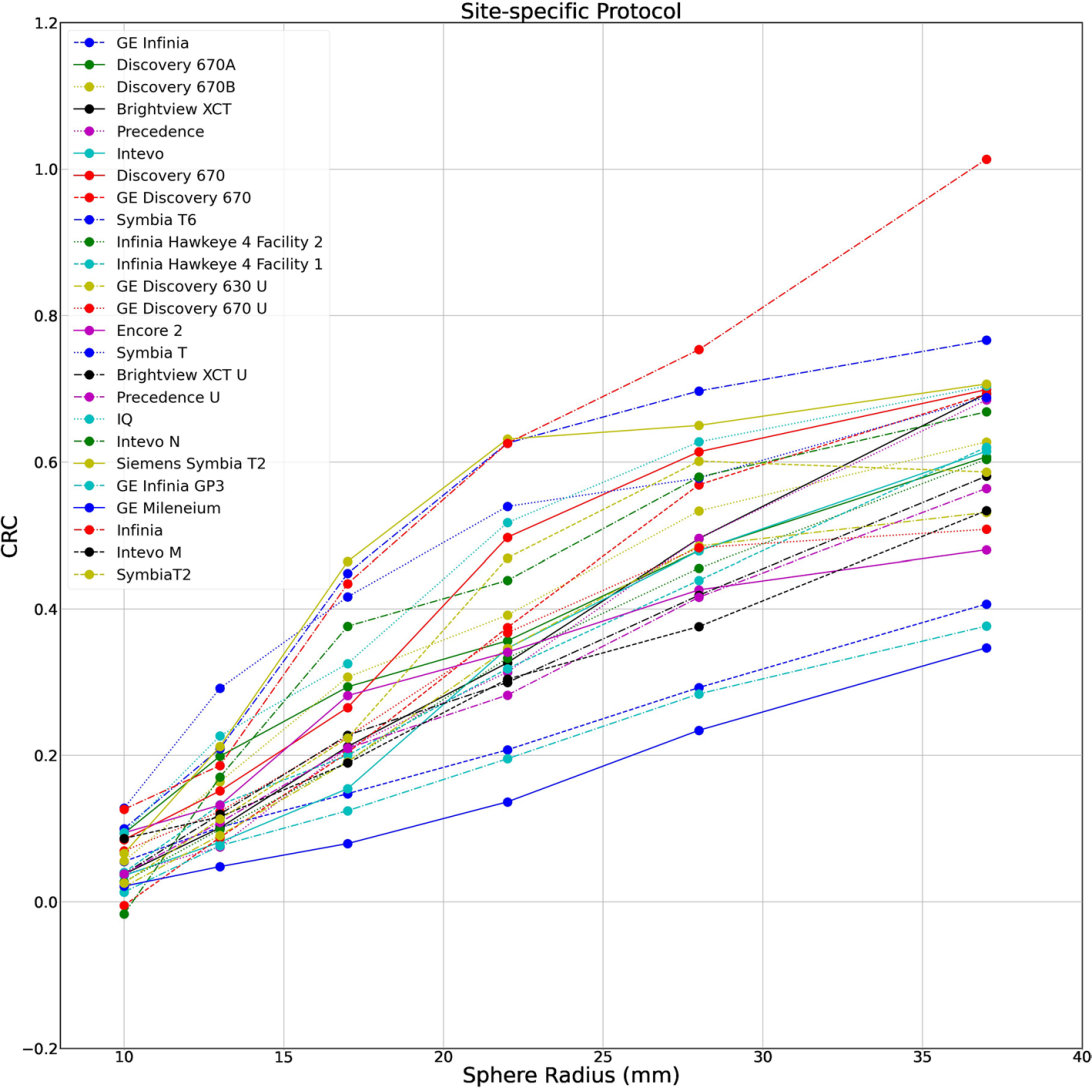
CRCs by sphere diameter for inserts 1 to 6 for site-specific acquisition and site-specific reconstruction protocols. A scatter plot of contrast recovery coefficient by sphere diameter for the 25 site-specific protocols

Figure 3 shows the distribution of sphere recoveries of all submitted data for the site-specific protocols. A large range in sphere recoveries was observed. CRC generally increased with sphere size, due to the larger impact of the partial volume effect on smaller structures. However, there were large differences for equal sphere sizes between centres/cameras. As an example, for the largest insert (37 mm diameter) the median CRC was 0.61 (0.53–0.69), and CRCs ranged from 0.35 to 1.01. The median S:B ratio was 5.28 (4.63–5.80) and S:B error was -2.64. A full overview of CRC for each insert, camera and protocol is given in Supplemental file table S3. Conducting attenuation correction and scatter correction yielded the highest average CRC, the median CRC for the 37 mm sphere for the subset of site-specific protocols applying attenuation and scatter correction was 0.68 (0.61–0.69). Comparing this with the subset where no attenuation correction and scatter correction was applied CRC was lowest with a larger variance, the median CRC for the 37 mm sphere being 0.51 (0.41–0.53).

**Fig. 4:**
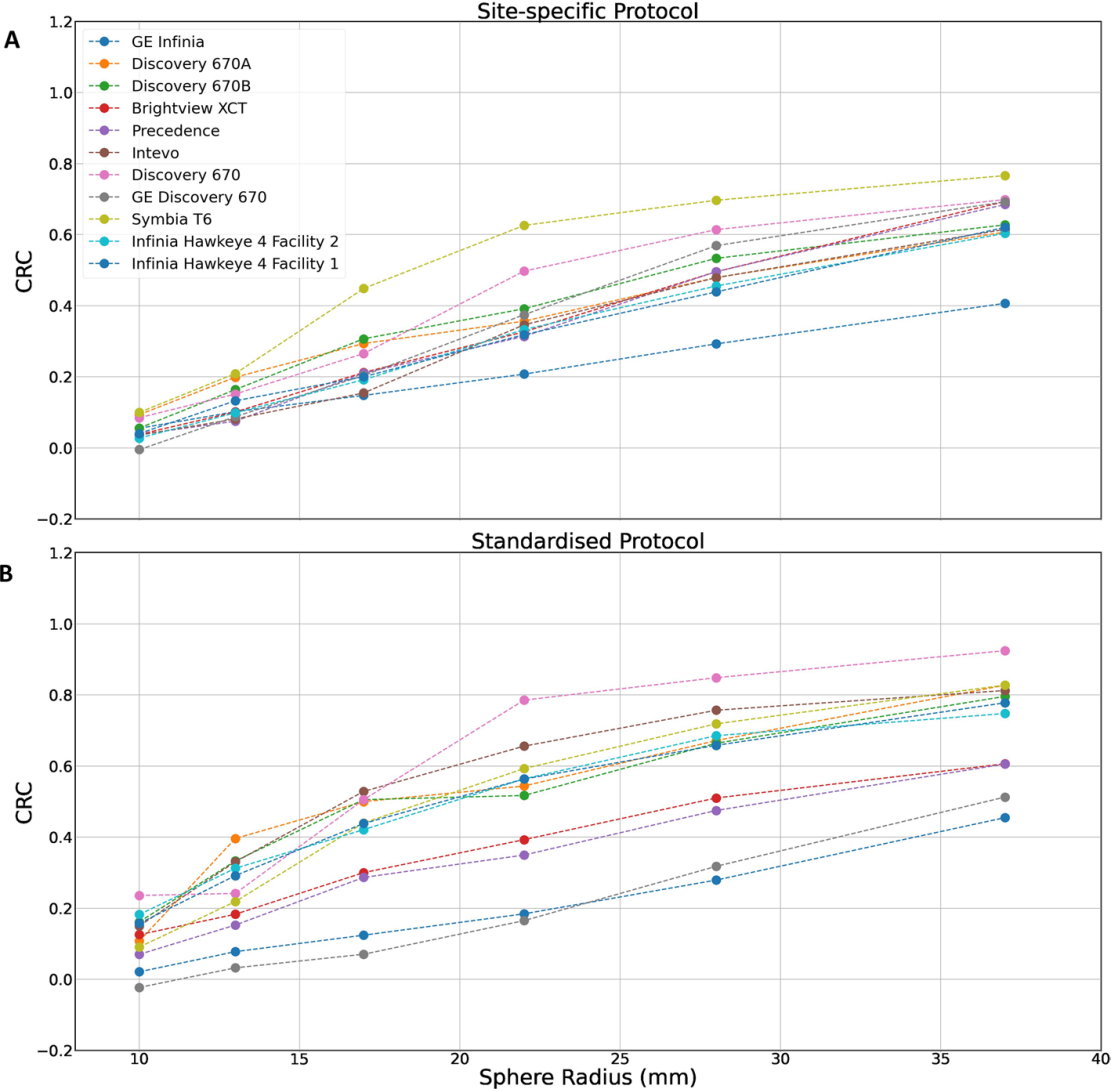
CRCs by sphere diameter for inserts 1 to 6 for site-specific acquisition and site-specific reconstruction protocols (paired subgroup) and for the standardised protocol. **A** A scatter plot of contrast recovery coefficient by sphere diameter for the 11 site-specific protocols which were included in the paired analysis. Protocols include both AC and SC, with the exception of the ‘GE Infinia’ protocol which includes AC and no SC. **B** A scatter plot of contrast recovery coefficient by sphere diameter for the 11 cameras that provided data acquired via the standardised protocol

Within the paired subgroup, the site-specific median CRC for the 37 mm sphere was 0.63 (0.61–0.69), and CRCs ranged from 0.41 to 0.77 (Table 5). The CRCs for the site-specific protocols within the paired subgroup are shown in Fig. 4A.

##### Standardised protocol

Data was available for 11 cameras and 11 protocols from 6 different centres. Figure 4B shows the CRCs obtained when the standardized acquisition and processing protocol was imposed.

Taking the largest sphere as an example, the median CRC was 0.78 (0.61–0.82), and the range was from 0.45 to 0.92. The median S:B ratio was 6.15 (5.19–6.85) and S:B error was -1.86. The CRC values from all (n = 25) site-specific protocols and the standardised protocol were significantly different (unpaired *t*-test *p* = 0.046). There was no significant difference between the CRC values of the site-specific paired subgroup (n = 11) and the standardised protocol (paired *t*-test *p* = 0.09).

A summary of measured CRC values for the site-specific (paired subgroup) and standardised protocols is given in Table 5.

**Fig. 5:**
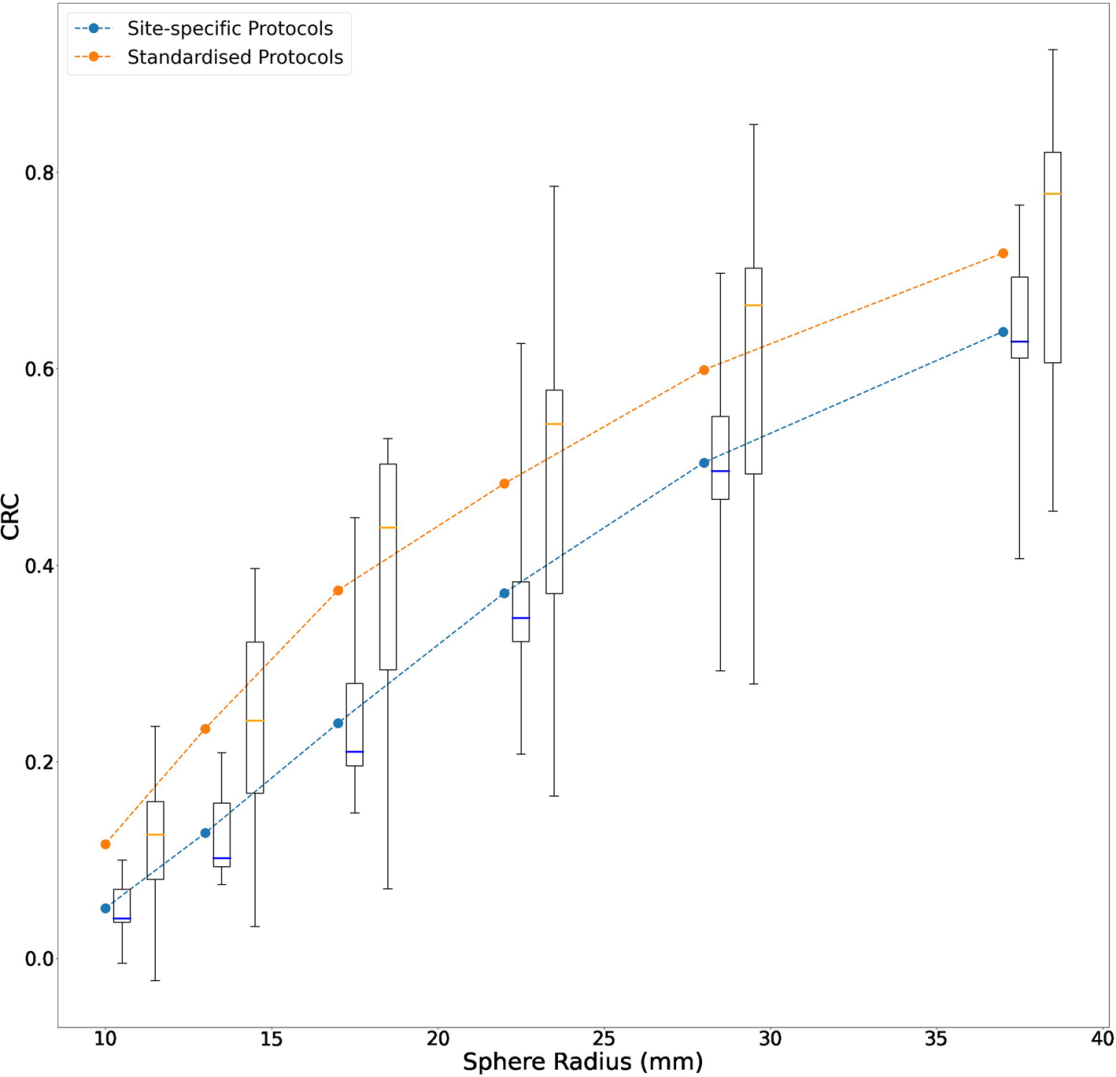
CRCs by sphere diameter for inserts 1 to 6 for site-specific protocols (paired subgroup) and the standardised protocol. The dashed lines represent the mean CRC across site-specific protocols (paired subgroup) and sites that provide data for the standardised protocol. Boxplots summarizing the CRC range for the site-specific (left) and standardised (right) protocols are included for each sphere diameter. The box represents the 25th to 75th percentile range and the central horizontal line represents the median value. The whiskers represent the range

Figure 5 highlights the variability in CRC for each sphere diameter using all site-specific protocols within the paired subgroup and the standardised protocol from all systems. The standardised protocol was associated with a higher CRC on average but greater variability.

## Discussion

The TARGET phantom Sub-Study aimed to evaluate inter-site variability in [^99m^Tc]Tc-MAA imaging and specifically, to report on variability in two dosimetric quantities (LSF and T:N due to technical and procedural differences between sites. To fully leverage the benefits of [^99m^Tc]Tc-MAA scout imaging, quantitative metrics derived from [^99m^Tc]Tc-MAA SPECT should be comparable between scanners, sites and studies. This study demonstrates that the use of key image corrections, specifically AC and SC, significantly reduced inter-system variability, whilst standardization of other reconstruction parameters (iterations, subsets and post-filtering) did not improve consistency.

Of the two metrics considered in this study, the LSF investigation exhibited better consistency between sites. Results showed the LSF was overestimated by approximately 8.8% using the site-specific protocols and the LSF IQR between different protocols was 9.6–10.1. Greater variability was noted in the tumour to normal tissue investigation, where CRC was demonstrated to vary substantially when site-specific imaging protocols were used. As an example, for the largest sphere in the NEMA IQ phantom, the CRC IQR was 0.5–0.7 and the two most differing sites recorded a CRC 0.35 and 1.01 respectively, meaning that if the same patient were scanned in two participating centres, the outcome in apparent tumour absorbed dose could differ by more than a factor of two. This demonstrates the potential variability that can be expected when sites use different imaging systems and methods.

An additional aim of the study was to investigate the impact of a harmonization strategy, involving implementation of a standardized imaging protocol. Firstly, considering the standardised protocol for the LSF investigation, the LSF as measured on the standardised protocol was underestimated by 5.0%, less than that noted for the site-specific protocols, however the IQR was not reduced (8.4–9.0). This indicates that the standardised protocol did not have a positive impact on variability but did improve accuracy. For the tumour dosimetry investigation, imposing the standardized protocol was evidenced to improve average performance. For the majority of centres (70%), the average CRCs of the largest sphere demonstrated a positive bias compared to site-specific protocols, and the S:B error was reduced, indicating accuracy was improved. The IQR however was not improved, as an example, the largest sphere CRC IQR increased from 0.16 (0.53–0.69) for the site-specific protocols where no specific corrections were imposed, to 0.21 (0.61–0.82) for the standardized protocol. It is evident, that implementing a single, standardised protocol does not necessarily reduce variability, as it is still necessary to account for the different properties of the collimators and the reconstruction algorithms for the various cameras. A key finding was that eliminating sources of possible variation in image corrections substantially improved inter-system quantification variability. For the subset of sites that provided both site-specific and standardised datasets, and who largely included attenuation and scatter correction as part of their site-specific protocols, the initial large variability in recovery coefficients was reduced. By removing inconsistency in only two parameters (AC and SC) the IQR in CRC for the 37 mm sphere halved from 0.2 to 0.1.

Our results demonstrate that technical factors have a non-negligible impact on [^99m^Tc]Tc-MAA image-based dosimetry and dose targets reported in multi-centre trials should be interpreted in this context. The benefits of defining specific dose targets when imaging practice remains markedly inconsistent is inherently limited. Efforts to define accurate dose thresholds must be matched by efforts to standardise imaging practice to maximise efficacy.

A simple step that may be taken to maximise consistency between centres, is to ask centres to perform key image corrections (i.e. scatter correction and attenuation correction). Since the site-specific subgroup of images that all applied AC and SC, were in fact more consistent than those obtained from the standardised protocol, this would suggest there is merit in investigating the implementation of image corrections and procedural guidelines but leaving the specific application to the discretion of individual centres with greater insight of their own imaging system.

Whilst the standardized protocol did require centres to perform these corrections, the protocol was prescriptive and did not leave much room for centres to optimise, leading to some inconsistent behaviours between different imaging systems. There were several factors that could introduce inconsistencies, for example, the standardized protocol stipulated that a 5 mm gaussian filter be used for post-filtering, however these filters may be implemented differently in the various reconstruction algorithms used by the respective vendors. Similarly, the energy window width in the standardized protocol was required to be 15% (140 keV ± 7.5%). Many centres had a default of 20% window width and thus, in order to adhere to the standardized protocol, the energy window settings were changed. For some scanners this would also require the scanner to be peaked. If the peaking procedure was skipped, this would result in poorer quality images. Finally, by tuning of the number of iterations, site-specific protocols typically balanced image noise and reconstruction time at the expense of a lower CRC. Since convergence rates vary between the various reconstruction algorithms for individual imaging systems, the standardized protocol utilized a relatively high number of iterations to assure full convergence and optimize the CRC. However, this resulted in high noise levels in several cases. A key takeaway therefore is that close agreement between sites via implementation of a standardised protocol for SPECT is only partly relevant, and potentially only pertinent for the acquisition parameters. Due to the different reconstruction algorithms and collimator specifics, a better harmonization approach could involve focusing on the CRC metric itself, and tuning reconstruction parameters accordingly leading to different settings for different cameras. Ideally this should be facilitated through a central entity to analyse the data, an approach which has been successfully demonstrated in the EARL initiative. A similar methodology, of implementing a standardised acquisition protocol and performing reconstruction centrally, has been successfully implemented for SPECT quantification of ^177^Lu [17] and ^99m^Tc [18] in phantoms.

This study has several limitations. Firstly, site specific differences in phantom preparation and activity dose measurement likely contributed to variability in the results, however as a specific preparation protocol was made available and the selected phantoms are standard in the field, this variance is expected to be small as compared to the objective of this investigation (i.e., the variability in acquisition and reconstruction). The LSF was inferred from two phantoms that were both homogeneously filled with activity and water, representing the ‘liver’ and ‘lungs’. In reality lungs are much less dense, activity is non-uniformly distributed, and the liver and lungs often overlap in the planar view, which in practice results in substantial overestimation of the LSF. For this reason many centres consider SPECT/CT as an alternative to planar imaging (alternative methods using SPECT/CT have been investigated and proven to be superior [19–21]). Finally, the standardized protocol for this Phantom sub-study was designed considering a system from a specific vendor, individual cameras from the various other imaging vendors implement different reconstruction algorithms and thus, a standardized protocol based on one imaging system was not capable of encompassing all scanner processes.

The dataset collected in this work, encompasses a wide range of system types and [^99m^Tc]Tc-MAA SPECT/CT imaging protocols and thus provides a representative insight into the large variability evident in the field. Future research should build on existing works [12] to establish an evidenced-based standardised practice of acquiring and reconstructing [^99m^Tc]Tc-MAA SPECT/CT images for the purpose of pre-treatment dosimetry. The harmonization of imaging procedures is now endorsed by several professional societies and organizations [22–24]. Much focus has been given to reducing variability of PET image quantification in multi-centre settings i.e. The EARL initiative [25], and more recently the AAPM launched a scheme to enhance consistency in ^90^Y Bremsstrahlung imaging, again based on quality control procedures using phantoms for standardisation [26]. However, as yet there is no standardisation programme for pre-treatment [^99m^Tc]Tc-MAA SPECT/CT dosimetry. Investment in an accreditation scheme similar to EARL for [^99m^Tc]Tc-MAA SPECT/CT dosimetry would be a valuable future endeavour to help advance the use of quantitative [^99m^Tc]Tc-MAA SPECT/CT imaging. In the interim, publications on dose–effect relationships and reported dose thresholds should comment on their centre-specific imaging factors (e.g., system type, protocol parameters, image quality as measured via CRC) so that other centres may put results into context before implementing clinically.

In conclusion, this study shows that quantification of [^99m^Tc]Tc-MAA SPECT/CT is feasible in a multi-centre phantom study, and high quality clinically relevant data can be obtained. Over the range of cameras and site-specific planar protocols investigated, comparable performance was noted in the lung shunt investigation, which suggests suitability for quantitative analysis of LSF in a scenario analogous to that of pre-treatment dosimetry work-up. Site-specific SPECT protocols included in this study were not capable of consistently reconstructing [^99m^Tc]Tc-MAA activity distributions and there were large differences in CRC between different protocols for the same size structure. By eliminating sources of difference in image corrections between protocols, variation in quantification was reduced. A subset of site-specific protocols that implemented key image corrections (AC and SC) had a reduced range compared to the full site-specific dataset. The standardised protocol did not improve consistency between sites in either the LSF investigation or tumour dosimetry investigation but did improve accuracy.

### Supplementary Information

Supplemental file 

## Data Availability

The datasets used and/or analysed during the current study are available from corresponding author on reasonable request.

## Abbreviations

^90^Y: Yttrium-90
HCC: Hepatocellular carcinoma
SPECT: Single photon emission computed tomography
CT: Computed tomography
SPECT/CT: Single photon emission computed tomography/computed tomography
[^99m^Tc]Tc-MAA: Technetium-99 macroaggregated albumin
TARGET: The TheraSphere™ Advanced Dosimetry Retrospective Global Study Evaluation in Hepatocellular Carcinoma Treatment
LSF: Lung shunt fraction
CRC: Contrast recovery coefficient
NEMA: National Electrical Manufacturing Association
IQ: Image quality
LEHR: Low Energy High Resolution collimators
DEW: Dual energy window
OSEM: Ordered subset expectation maximization
AC: Attenuation correction
SC: Scatter correction
IQR: Interquartile range
ROI: Region of interest
VOI: Volume of interest
S:B: Sphere to background ratio
RR: Resolution recovery
T:N: Tumour to normal tissue ratio

## References

[1] Salem R, Padia SA, Lam M, Bell J, Chiesa C, Fowers K (2019). Clinical and dosimetric considerations for Y90: recommendations from an international multidisciplinary working group. Eur J Nucl Med Mol Imaging.

[2] Garin E, Rolland Y, Pracht M, Le Sourd S, Laffont S, Mesbah H (2017). High impact of macroaggregated albumin-based tumour dose on response and overall survival in hepatocellular carcinoma patients treated with (90) Y-loaded glass microsphere radioembolization. Liver Int.

[3] Lam M, Garin E, Maccauro M, Kappadath SC, Sze DY, Turkmen C (2022). A global evaluation of advanced dosimetry in transarterial radioembolization of hepatocellular carcinoma with yttrium-90: the TARGET study. Eur J Nucl Med Mol Imaging.

[4] Chiesa C, Mira M, Maccauro M, Romito R, Spreafico C, Sposito C (2012). A dosimetric treatment planning strategy in radioembolization of hepatocarcinoma with 90Y glass microspheres. Q J Nucl Med Mol Imaging.

[5] Garin E, Tselikas L, Guiu B, Chalaye J, Edeline J, de Baere T (2021). Personalised versus standard dosimetry approach of selective internal radiation therapy in patients with locally advanced hepatocellular carcinoma (DOSISPHERE-01): a randomised, multicentre, open-label phase 2 trial. Lancet Gastroenterol Hepatol.

[6] Kao YH, Steinberg JD, Tay YS (2013). Post-radioembolization yttrium-90 PET/CT - part 2: dose-response and tumor predictive dosimetry for resin microspheres.. EJNMMI Res.

[7] Garin E, Lenoir L, Edeline J, Laffont S, Mesbah H, Poree P (2013). Boosted selective internal radiation therapy with 90Y-loaded glass microspheres (B-SIRT) for hepatocellular carcinoma patients: a new personalized promising concept. Eur J Nucl Med Mol Imaging.

[8] Pacilio M, Ferrari M, Chiesa C (2016). Impact of SPECT corrections on 3D-dosimetry for liver transarterial radioembolization using the patient relative calibration methodology. Med Phys.

[9] Chiesa C, Mira M, Maccauro M (2015). Radioembolization of hepatocarcinoma with (90)Y glass microspheres: development of an individualized treatment planning strategy based on dosimetry and radiobiology. Eur J Nucl Med Mol Imaging.

[10] Botta F, Ferrari M, Chiesa C (2018). Impact of missing attenuation and scatter corrections on 99m Tc-MAA SPECT 3D dosimetry for liver radioembolization using the patient relative calibration methodology: A retrospective investigation on clinical images. Med Phys.

[11] van der Velden S, Dietze MMA, Viergever MA, de Jong HWAM (2019). Fast technetium-99m liver SPECT for evaluation of the pretreatment procedure for radioembolization dosimetry. Med Phys.

[12] Lam M, Garin E, Maccauro M, Kappadath C, Sze D, Turkmen C, Cantasdemir M, Haste P, Herrmann K, Alsuhaibani H, Dreher M, Fowers K, Salem R (2022). A global evaluation of advanced dosimetry in transarterial radioembolization of hepatocellular carcinoma with yttrium-90: the TARGET study. EJNMMI.

[13] Chiesa C, Sjogreen-Gleisner K, Walrand S, Strigari L, Flux G, Gear J (2021). EANM dosimetry committee series on standard operational procedures: a unified methodology for 99mTc-MAA pre- and 90Y peri-therapy dosimetry in liver radioembolization with 90Y microspheres. EJNMMI Physics.

[14] Perkins A, Stearns C, Chapman J, Kolthammer J, Williams J, Casey M, et al. NEMA NU-2 standards publication performance measurements of positron emission tomographs. National Electrical Manufacturers Association; 2007.

[15] Otsu M (1979). A threshold selection method from gray-level histograms. IEEE Trans Syst Man Cybern.

[16] Chang LT (1978). A method for attenuation correction in radionuclide computed tomography. IEEE Trans Nucl Sci.

[17] Peters S, Meyer Viol S, van der Werf N, de Jong N, van Velden F, Meeuwis A (2020). Variability in lutetium-177 SPECT quantification between different state-of-the-art SPECT/CT systems. EJNMMI Phys.

[18] Peters SMB, van der Werf NR, Segbers M (2019). Towards standardization of absolute SPECT/CT quantification: a multi-center and multi-vendor phantom study. EJNMMI Phys.

[19] Elsayed M, Cheng B, King M, Seth I, Brandon B, Schuster D (2021). Comparison of Tc-99m MAA planar versus SPECT/CT imaging for lung shunt fraction evaluation prior to Y-90 radioembolization: are we overestimating lung shunt fraction?. Cardiovasc Intervent Radiol.

[20] Kappadath SC, Lopez BP, Salem R, Lam MG (2021). Lung shunt and lung dose calculation methods for radioembolization treatment planning. Q J Nucl Med Mol Imaging.

[21] Lopez B, Mahvash A, Lam MGEH, Kappadath SC (2019). Calculation of lung mean dose and quantification of error for 90 Y-microsphere radioembolization using 99m Tc-MAA SPECT/CT and diagnostic chest CT. Med Phys.

[22] Lasnon C, Desmonts C, Quak E, Gervais R, Do P, Dubos-Arvis C (2013). Harmonizing SUVs in multicentre trials when using different generation PET systems: prospective validation in non-small cell lung cancer patients. Eur J Nucl Med Mol Imaging.

[23] Boellaard R, Delgado-Bolton R, Oyen WJG, Giammarile F, Tatsch K, Eschner W (2014). FDG PET/CT: EANM procedure guidelines for tumour imaging: version 2.0. Eur J Nucl Med Mol Imaging.

[24] Delbeke D, Coleman RE, Guiberteau MJ, Brown ML, Royal HD, Siegel BA (2006). Procedure guideline for tumor imaging with 18FFDG PET/CT 1.0. J Nucl Med.

[25] Kaalep A, Sezra T, Oyen W, Krause B, Chiti A, Liu Y, Boellaard R (2018). EANM/EARL FDG-PET/CT accreditation—summary results from the first 200 accredited imaging systems. Eur J Nucl Med Mol Imaging.

[26] AAPM. Standardizing imaging and reconstruction protocols for quantitative SPECT/CT post yttrium-90 microspheres delivery (SIRP. [Online]. American Association of Physicists in Medicine. 2022; Available at: https://www.aapm.org/GrandChallenge/SIRPRISE/. Accessed 1 May 2023.

