# Supplementary material for: An international phantom study of inter-site variability in Technetium-99m image quantification: analyses from the TARGET radioembolization study
Source: EJNMMI Phys. 2024 May 29;11:46. doi: 10.1186/s40658-024-00647-x (PMC11136909; doi:10.1186/s40658-024-00647-x)
Supplement: Supplementary file 1 — Supplemental file [file 40658_2024_647_MOESM1_ESM.docx]

# TABLES (SUPPLEMENT)

# Table 1. Injected activity data for phantoms prepared at all sites (NB. Centres 840050 and 840098 prepared more than one phantom).

| **Site ID** | **250011** | **840047** | **840048** | **528001** | **840050** | **840050** | **840098** | **840098** | **840098** |
| --- | --- | --- | --- | --- | --- | --- | --- | --- | --- |
| Injected Activity (MBq) Syringe 1* at reference time | 278.00 | 225.38 | 247.14 | 239.00 | 255.76 | 240.67 | 134.13 | 132.50 | 268.60 |
| Injected Activity (MBq) Syringe 2** at reference time | 339.69 | 263.24 | 305.94 | 292.08 | 294.04 | 300.24 | 322.43 | 329.30 | 326.93 |
| Injected Activity (MBq) Syringe 3*** at reference time | 37.53 | 27.78 | 31.06 | 26.42 | 31.08 | 33.05 | 33.51 | 34.86 | 33.31 |
| Volume total inserts (ml) | 47.84 | 47.84 | 47.84 | 47.84 | 47.84 | 47.84 | 47.84 | 47.84 | 47.84 |
| Volume of stock solution (ml) | 1000 | 1000 | 1000 | 1000 | 1000 | 1000 | 501 | 506 | 1006 |
| Volume of background (ml) | 9700 | 9700 | 9700 | 9700 | 9700 | 9700 | 9700 | 9700 | 9700 |
| Activity in NEMA (MBq) | 352.99 | 274.03 | 317.76 | 303.51 | 306.27 | 311.75 | 335.24 | 341.83 | 339.70 |
| Activity in cylinder (MBq) | 37.53 | 27.78 | 31.06 | 26.42 | 31.08 | 33.05 | 33.51 | 34.86 | 33.31 |
| LSF (%) | 9.6 | 9.2 | 8.9 | 8.0 | 9.2 | 9.6 | 9.1 | 9.3 | 8.9 |
| Sphere:Background | 7.94 | 8.30 | 7.84 | 7.94 | 8.44 | 7.78 | 8.05 | 7.71 | 7.92 |

| **Site ID** | **756001** | **276010** | **840017** | **840049** | **840051** | **792001** | **792002** | **380010** |
| --- | --- | --- | --- | --- | --- | --- | --- | --- |
| Injected Activity (MBq) Syringe 1* at reference time | 136.60 | 230.78 | 244.68 | 239.40 | 240.94 | 236.69 | 241.90 | 265.88 |
| Injected Activity (MBq) Syringe 2** at reference time | 291.50 | 286.49 | 288.58 | 287.90 | 316.99 | 305.70 | 294.15 | 330.22 |
| Injected Activity (MBq) Syringe 3*** at reference time | 34.77 | 26.63 | 30.95 | 29.60 | 32.48 | 30.88 | 28.72 | 36.98 |
| Volume total inserts (ml) | 47.84 | 47.84 | 47.84 | 47.84 | 47.84 | 47.84 | 47.84 | 47.84 |
| Volume of stock solution (ml) | 500 | 1000 | 1000 | 1000 | 1000 | 1000 | 1000 | 1000 |
| Volume of background (ml) | 9340 | 9700 | 9700 | 9700 | 9700 | 9700 | 9700 | 9700 |
| Activity in NEMA (MBq) | 304.57 | 297.53 | 300.28 | 299.35 | 328.51 | 317.03 | 305.72 | 342.94 |
| Activity in cylinder (MBq) | 34.77 | 26.63 | 30.95 | 29.60 | 32.48 | 30.88 | 28.72 | 36.98 |
| LSF (%) | 10.2 | 8.2 | 9.3 | 9.0 | 9.0 | 8.9 | 8.6 | 9.7 |
| Sphere:Background | 8.75 | 7.81 | 8.22 | 8.07 | 7.37 | 7.51 | 7.98 | 7.81 |

*Syringe 1 contained approximately 240MBq in 1ml saline, this was added to 1000ml saline to create a stock solution which was injected into the 6 spherical inserts. **Syringe 2 contained approximately 290MBq in 1ml saline, this was injected into the background of the NEMA phantom after filling it with water. ***Syringe 3 contained approximately 30MBq in 1ml saline, this was injected into the cylindrical phantom after filling it with water.

# Table 2. Overview of site-specific protocols for each centre

| **phase** | **Parameter** | **250011** | **840047** | **840048a** | **840048b** | **528001** |
| --- | --- | --- | --- | --- | --- | --- |
| energy and planar | Emission energy window (range, center) | 140 keV /15% | 140 keV / +-10% | 140 keV /20% (126.4/154.5) | 140 keV /20% | 140 keV /15% |
|  | Scatter window | 120.5 keV/15% | 120 +- 5% | None | None | 120.5 keV/15% |
|  | Number of acquisitions | 1 | 2 | 1 | 1 | 2 |
|  | Patient position (Feet first, Head first) | FFS | FFS | FFS | FFS | FFS |
|  | Acquisition time per frame (min) | N/A see note | NA | N/A | N/A | 5 |
|  | Zoom | 1 | 1 | 0.92 | 0.92 | 1 |
|  | Matrix size | 256 x 256 | 256x1024 | 256x1024 | 256x1024 | 256x256 |
|  | Additional information: | counts stop: 800 kc | continous motion | continous WB | continous WB | 2x acquisitions: 1x centred lung, 1x centred liver |
| SPECT | Matrix size | 128 x 128 | 128x128 | 256x256 | 256x256 | 128x128x128 |
| Acquisition | Zoom | 1 | 1 | 1.1 | 1.1 | 1.23 |
|  | Number of heads | 2 | 2 | 2 | 2 | 2 |
|  | Number of projections in total | 64 i.e. 32 per head | 60, i.e. 30 per head | 60, i.e. 30 per head | 60, i.e. 30 per head | 120, i.e. 60 per head |
|  | Time per projection [sec] | 25 | 30 | 30 | 30 | 20 |
|  | Orbit (circular, non-circular close orbit) | Non circular | non-circular close orbit | non-circular close orbit | non-circular close orbit | non-circular close orbit |
| SPECT | Type of reconstruction (OS-EM, FBP, etc.) | OSEM | OS-EM | OS-EM | OS-EM | OS-EM Flash3D |
| Recon | Matrix | 128 x 128 | 128x128x128 | 256x256x256 | 256x256x256 | 128x128x128 |
|  | Iterations (OSEM) | 5 | 2 | 2 | 2 | 6 |
|  | Subsets (OSEM) | 8 | 10 | 10 | 10 | 8 |
|  | Slice thickness | 4.51 mm | 4.418 | 2.01 | 2.01 | 3.9 |
|  | Attenuation correction (yes or no) – method | Yes – CT-based attenuation map | Yes – CT-based attenuation map | Yes - Chang | Yes – CT-based attenuation map | Yes – CT-based attenuation map |
|  | Scatter correction method (none, DEW, etc.) | DEW | None | None | None | DEW |
|  | Other (partial volume effect, etc.) | PSF | None | None | None | PSF |
|  | Filtering | **4 mm Gaussian** | HANN | Butterworth | Butterworth | 5mm Gaussian |

| **phase** | **parameter** | **840050a** | **840050b** | **840098a** | **840098b** | **840098c** |
| --- | --- | --- | --- | --- | --- | --- |
| energy and planar | Emission energy window (range, center) | 140 keV + 15% | 140 keV/ 15% | 140.5 +- 10% (126.45/154.95 | 140.5 +- 10% (126.45/154.95 | 140.5 +- 10% (126.46 - 154.56) |
|  | Scatter window | None for planar, DEW 15% for SPECT | None for planar, DEW 15% for SPECT | None for planar 114/126 | None for planar 114/126 | None |
|  | Number of acquisitions | 2 | 2 | 2 | 2 | 2 |
|  | Patient position (Feet first, Head first) | Feet | Feet | feet first | feet first | FFS |
|  | Acquisition time per frame (min) | 5 | 5 | 5 | 5 | 5 |
|  | Zoom | 1.45 | 1.45 | 1 | 1 | 1 |
|  | Matrix size | 256X256 | 256X256 | 256x256 | 256x256 | 256x256 |
|  | Additional information: | 2x acquisitions: 1x centred lung, 1x centred liver | 2x acquisitions: 1x centred lung, 1x centred liver |  |  |  |
| SPECT | Matrix size | 128x128 | 128x128 | 128x128 | 128x128 | 128x128 |
| Acquisition | Zoom | 1 | 1 | 1 | 1 | 1 |
|  | Number of heads | 2 | 2 | 2 | 2 | 2 |
|  | Number of projections in total | 64, i.e. 32 per head | 64, i.e. 32 per head | 120 | 120 | 128 |
|  | Time per projection [sec] | 35 | 70 | 10 | 10 | 10 |
|  | Orbit (circular, non-circular close orbit) | non-circular step and shoot | non-circular step and shoot | non-circular close | non-circular close | non-circular orbit |
| SPECT | Type of reconstruction (OS-EM, FBP, etc.) | FLASH3D | FLASH3D | OSEM | OSEM | OSEM - ASTONISH |
| Recon | Matrix | 128x128x128 | 128x128x128 | 128x128 | 128x128 | 130x90x90 |
|  | Iterations (OSEM) | 10 | 10 | 2 | 2 | 2 |
|  | Subsets (OSEM) | 8 | 8 | 10 | 10 | 16 |
|  | Slice thickness | N/A | N/A | 4.42 | 4.42 | 4.66 |
|  | Attenuation correction (yes or no) – method | CTAC convolution & 3DOSEM (10i, 8s) | CTMUAC convolution & 3DOSEM 10i, 8 s) | Yes (CT) | Yes (CT) | yes - ? |
|  | Scatter correction method (none, DEW, etc.) | DEW | DEW | DEW | DEW | ESSE |
|  | Other (partial volume effect, etc.) | none | none | Resolution recovery | Resolution recovery | RR (automatic with astonish) |
|  | Additional information: |  |  |  |  |  |
|  | Filtering | 6 mm Gaussian | 6 mm Gaussian | Butterworth 0.48/10 | Butterworth 0.48/10 | Hanning 1.0 |
| **phase** | **parameter** | **840098d** | **840098e** | **840098f** | **756001a** | **756001b** |
| energy and planar | Emission energy window (range, center) |  | 126.46 - 154.56 |  | 140.5 /15% (130-151 ) | 140.5 /20% |
|  | Scatter window |  | None |  | 120 /15% (111-129)- not for planar | DEW 10%- not for planar |
|  | Number of acquisitions |  | 2 |  | 1 | 1 |
|  | Patient position (Feet first, Head first) |  | FFS |  | FFS | FFS |
|  | Acquisition time per frame (min) |  | 5 |  | 2 | 2 |
|  | Zoom |  | 1 |  | 1 | 1 |
|  | Matrix size |  | 256x256 |  | 256x256 | 256x256 |
|  | Additional information: |  | 2x acquisitions: 1x centred lung, 1x centred liver |  | single acquisition | single acquisition |
| SPECT | Matrix size |  | 128x128 |  | 256x256 | 128x128 |
| Acquisition | Zoom |  | 1 |  | 1 | 1 |
|  | Number of heads |  | 2 |  | 2 | 2 |
|  | Number of projections in total |  | 128 |  | 120 | 120 |
|  | Time per projection [sec] |  | 10 |  | 20 | 15 |
|  | Orbit (circular, non-circular close orbit) |  | non-cirular orbit |  | non-circ orbit | non-circ orbit |
| SPECT | Type of reconstruction (OS-EM, FBP, etc.) | OSEM | OSEM - ASTONISH | OSEM | OSCG Flash 3D | OSEM |
| Recon | Matrix | 130x90x90 | 128x128x128 | 128x128x128 | 256x256x256 | 128x128x128 |
|  | Iterations (OSEM) | 3 | 2 | 3 | 16 | 2 |
|  | Subsets (OSEM) | 8 | 16 | 8 | 1 | 10 |
|  | Slice thickness | 4.66 |  |  |  | 4.42 |
|  | Attenuation correction (yes or no) – method | yes - ? | Yes CT | Yes CT | Yes CT | Yes CT |
|  | Scatter correction method (none, DEW, etc.) | ESSE | ESSE | ESSE | DEW | DEW |
|  | Other (partial volume effect, etc.) |  | RR | None | RR | RR |
|  | Additional information: |  |  |  |  |  |
|  | Filtering | Butterworth 0.6 | Hanning loop filter | Butterworth 0.6 | 10 mm Gaussian | None |

| **phase** | **Parameter** | **276010** | **840017** | **840049** | **840051** | **792001a** |
| --- | --- | --- | --- | --- | --- | --- |
| energy and planar | Emission energy window (range, center) | 140 keV/ 15%: | 140 keV/ 15%: | 140 keV /15% | 140 keV /20% | 140 keV /20% |
|  | Scatter window | None for planar, DEW 15% for SPECT (107.94 ; 128.8) | None | None for planar; DEW for spect 120.5 keV/15% | planar none; 120/10% | no |
|  | Number of acquisitions | 1 | 2 ANT CHEST LIVER | 2 | 1 WB | 1 WB |
|  | Patient position (Feet first, Head first) | Feet | FFS | Feet | FFS | FFS |
|  | Acquisition time per frame (min) | WB 200cm with 15cm/min | 5 | 7 | 10 min 9cm/min | 10 cm/min |
|  | Zoom | 1 | 1.455 | 1.23 | 1 | 1 |
|  | Matrix size | 256X1024 | 128x128 | 128x128 |  | 256x1024 |
|  | Additional information: | Whole body |  | 2x acquisitions: 1x centred lung, 1x centred liver |  |  |
| SPECT | Matrix size | 128x128x128 | 128x128 | 128x128x128 | 64x64 | 128x128 |
| Acquisition | Zoom | 1 | 1.23 | 1 | 1 | 1 |
|  | Number of heads | 2 | 2 | 2 | 2 | 2 |
|  | Number of projections in total | 128, i.e. 64 per head | 80, i.e. 40 per head | 128, i.e. 64 per head | 120, i.e. 60 per head | 120, i.e. 60 per head |
|  | Time per projection [sec] | 15 | 12 | 16 | 15 | 20 |
|  | Orbit (circular, non-circular close orbit) | non-circular close orbit | - | non-circular close orbit | non-circular close orbit | circular |
| SPECT | Type of reconstruction (OS-EM, FBP, etc.) | FLASH3D | FBP | FLASH3D | OS-EM | OS-EM |
| Recon | Matrix | 128x128x128 | 128x128 | 128x128x128 | 64x64 | 128 |
|  | Iterations (OSEM) | 8 |  | 8 | 3 | 2 |
|  | Subsets (OSEM) | 4 |  | 16 | 15 | 10 |
|  | Slice thickness | N/A |  | 4.8mm |  | 4.41mm |
|  | Attenuation correction (yes or no) – method | CT-based attenuation map | Yes chang | CT-based attenuation map | CT-based attenuation map | no |
|  | Scatter correction method (none, DEW, etc.) | DEW | None | DEW | MC based or DEW | no |
|  | Other (partial volume effect, etc.) | none | None | RR | PSF | PSF |
|  | Filtering |  | BTW 0.40 | 5mm Gaussian | 1.35cm Gaussian | Butterworth 0.48 |

| **phase** | **Parameter** | **792001b** | **792002** | **380010a** | **380010b** |  |
| --- | --- | --- | --- | --- | --- | --- |
| energy and planar | Emission energy window (range, center) | 140 keV /20% | 140.5 keV /20% | 140 keV /15% | 140 keV /20% |  |
|  | Scatter window | no | DEW 20% | DEW 15% | DEW 15% |  |
|  | Number of acquisitions |  | WB |  |  |  |
|  | Patient position (Feet first, Head first) | FFS | FFS |  |  |  |
|  | Acquisition time per frame (min) |  | 13 cm/min |  |  |  |
|  | Zoom | 1 | 1 |  |  |  |
|  | Matrix size | 256x1024 |  |  |  |  |
|  | Additional information: |  |  |  |  |  |
| SPECT | Matrix size | 128x128 | 128x128 | 256x256 |  |  |
| Acquisition | Zoom | 1 | 1 | 1 |  |  |
|  | Number of heads | 2 | 2 | 2 |  |  |
|  | Number of projections in total | 120, i.e. 60 per head | 60 | 120 |  |  |
|  | Time per projection [sec] | 20 | 20 | 20 |  |  |
|  | Orbit (circular, non-circular close orbit) | circular | not specified | non-circular close orbit |  |  |
| SPECT | Type of reconstruction (OS-EM, FBP, etc.) | FBP | OS-EM | OSCG (XSPECT recon) | 3D OSEM |  |
| Recon | Matrix | 128 | 128 | 256 | 128 |  |
|  | Iterations (OSEM) |  | 2 | 24 | 8 |  |
|  | Subsets (OSEM) |  | 10 | 2 | 8 |  |
|  | Slice thickness | 4.52 | N/A | 1.95 | 4.42 |  |
|  | Attenuation correction (yes or no) – method | no | Yes - CT | Yes - CT | Yes - CT |  |
|  | Scatter correction method (none, DEW, etc.) | no | DEW | DEW |  |  |
|  | Other (partial volume effect, etc.) | no | none | Resolution recovery | Resolution recovery |  |
|  | Filtering | Butterworth 0.28 | Butterworth 0.48 | 10mm |  |  |

**Table 3. Overview of CRCs for each site-specific protocol**

| **Centre** | **Camera** | **Protocol Name** | **Protocol Parameters** | **Insert 1 (10mm)** | **Insert 2**  **(13mm)** | **Insert 3**  **(17mm)** | **Insert 4**  **(22mm)** | **Insert 5**  **(28mm)** | **Insert 6**  **(37mm)** |
| --- | --- | --- | --- | --- | --- | --- | --- | --- | --- |
| 840047 | GE Infinia | Site-specific 1 | Site-specific - AC, No SC | 0.055 | 0.102 | 0.148 | 0.208 | 0.293 | 0.407 |
| 840048 | GE Discovery 630 | Site-specific 1 | Site-specific - AC, No SC | 0.020 | 0.091 | 0.191 | 0.346 | 0.486 | 0.531 |
| 840048 | GE Discovery 670 | Site-specific 1 | Site-specific - AC, No SC | 0.070 | 0.123 | 0.226 | 0.367 | 0.483 | 0.508 |
| 840017 | Encore 2 | Site-specific 1 | Site-specific - AC, No SC | 0.094 | 0.132 | 0.282 | 0.341 | 0.426 | 0.480 |
| 528001 | Symbia T | Site-specific 1 | Site-specific - AC and SC | 0.128 | 0.292 | 0.417 | 0.539 | 0.578 | 0.688 |
| 840098 | Discovery 670A | Site-specific 1 | Site-specific - AC and SC | 0.094 | 0.199 | 0.294 | 0.356 | 0.479 | 0.607 |
| 840098 | Discovery 670B | Site-specific 1 | Site-specific - AC and SC | 0.056 | 0.164 | 0.307 | 0.391 | 0.533 | 0.627 |
| 840098 | Brightview XCT | Site-specific 1 | Site-specific - AC and SC | 0.038 | 0.101 | 0.212 | 0.326 | 0.496 | 0.693 |
| 840098 |  | Site-specific 2 | Site-specific - AC and SC | 0.038 | 0.120 | 0.228 | 0.299 | 0.419 | 0.581 |
| 840098 | Precedence | Site-specific 1 | Site-specific - AC and SC | 0.037 | 0.075 | 0.210 | 0.313 | 0.496 | 0.685 |
| 840098 |  | Site-specific 2 | Site-specific - AC and SC | 0.038 | 0.109 | 0.210 | 0.282 | 0.416 | 0.564 |
| 756001 | Intevo | Site-specific 1 | Site-specific - AC and SC | 0.036 | 0.081 | 0.155 | 0.346 | 0.479 | 0.615 |
| 756001 | Discovery 670 | Site-specific 1 | Site-specific - AC and SC | 0.085 | 0.152 | 0.265 | 0.497 | 0.614 | 0.699 |
| 276010 | IQ | Site-specific 1 | Site-specific - AC and SC | 0.094 | 0.227 | 0.325 | 0.517 | 0.628 | 0.704 |
| 792002 | GE Discovery 670 | Site-specific 1 | Site-specific - AC and SC | -0.005 | 0.088 | 0.204 | 0.375 | 0.569 | 0.693 |
| 840049 | Symbia T6 | Site-specific 1 | Site-specific - AC and SC | 0.010 | 0.209 | 0.448 | 0.626 | 0.697 | 0.766 |
| 840050 | Intevo | Site-specific 1 | Site-specific - AC and SC | -0.017 | 0.170 | 0.376 | 0.439 | 0.580 | 0.669 |
| 840051 | Infinia Hawkeye 4 | Site-specific 1 | Site-specific - AC and SC | 0.040 | 0.133 | 0.200 | 0.318 | 0.439 | 0.620 |
| 840051 |  | Site-specific 2 | Site-specific - AC and SC | 0.027 | 0.099 | 0.192 | 0.333 | 0.455 | 0.604 |
| 250011 | Siemens Symbia T2 | Site-specific 1 | Site-specific - AC and SC | 0.066 | 0.212 | 0.465 | 0.631 | 0.650 | 0.707 |
| 792001 | GE Infinia GP3 | Site-specific 1 | Site-specific - No AC, No SC | 0.013 | 0.077 | 0.125 | 0.195 | 0.284 | 0.377 |
| 792001 | GE Mileneium | Site-specific 1 | Site-specific - No AC, No SC | 0.021 | 0.048 | 0.080 | 0.137 | 0.234 | 0.347 |
| 380010 | Infinia | Site-specific 1 | Site-specific - No AC, SC | 0.126 | 0.186 | 0.434 | 0.626 | 0.754 | 1.014 |
| 380010 | Intevo | Site-specific 1 | Site-specific - No AC, SC | 0.087 | 0.116 | 0.190 | 0.304 | 0.376 | 0.534 |
| 840050 | SymbiaT2 | Site-specific 1 | Site-specific - No AC, SC | 0.026 | 0.113 | 0.224 | 0.469 | 0.601 | 0.587 |
